# Peripheral Injection of SB203580 Inhibits the Inflammatory-Dependent Synthesis of Proinflammatory Cytokines in the Hypothalamus

**DOI:** 10.1155/2014/475152

**Published:** 2014-06-04

**Authors:** Andrzej P. Herman, Agata Krawczyńska, Joanna Bochenek, Hanna Antushevich, Anna Herman, Dorota Tomaszewska-Zaremba

**Affiliations:** ^1^Polish Academy of Sciences, The Kielanowski Institute of Animal Physiology and Nutrition, 05-110 Jabłonna, Poland; ^2^The Academy of Cosmetics and Health Care, 13 Podwale Street, 00-252 Warsaw, Poland

## Abstract

The study was designed to determine the effects of peripheral injection of SB203580 on the synthesis of interleukin- (IL-) 1**β**, IL-6, and tumor necrosis factor (TNF) **α** in the hypothalamus of ewes during prolonged inflammation. Inflammation was induced by the administration of lipopolysaccharide (LPS) (400 ng/kg) over 7 days. SB203580 is a selective ATP-competitive inhibitor of the p38 mitogen-activated protein kinase (MAPK), which is involved in the regulation of proinflammatory cytokines IL-1**β**, IL-6 and TNF**α** synthesis. Intravenous injection of SB203580 successfully inhibited (*P* < 0.01) synthesis of IL-1**β** and reduced (*P* < 0.01) the production of IL-6 in the hypothalamus. The p38 MAPK inhibitor decreased (*P* < 0.01) gene expression of TNF**α** but its effect was not observed at the level of TNF**α** protein synthesis. SB203580 also reduced (*P* < 0.01) LPS-stimulated IL-1 receptor type 1 gene expression. The conclusion that inhibition of p38 MAPK blocks LPS-induced proinflammatory cytokine synthesis seems to initiate new perspectives in the treatment of chronic inflammatory diseases also within the central nervous system. However, potential proinflammatory effects of SB203580 treatment suggest that all therapies using p38 MAPK inhibitors should be introduced very carefully with analysis of all expected and unexpected consequences of treatment.

## 1. Introduction

Hypothalamus is the brain region responsible for integration and processing of signals from endocrine, nervous, and immune systems. It is well known that inflammatory states influence the activity of neurons located in different hypothalamic nuclei and affect centrally regulated processes such as thermogenesis [1], food intake [2], reproduction [3, 4], and circadian rhythms of rest-activity and sleep [5]. Among the most important mediators that transmit the inflammatory signals from the periphery into brain parenchyma are proinflammatory cytokines. It is well established that inflammatory processes raise the concentration of proinflammatory cytokines such as interleukin- (IL-) 1*β*, IL-6, and tumor necrosis factor (TNF) *α* in the peripheral blood [6, 7]. During the development of inflammatory response, the increased concentration of proinflammatory cytokines is observed both in the cerebrospinal fluid and in brain parenchyma [6, 8, 9]. The origin of these proinflammatory cytokines in central nervous system seems to be differentiated: the peripheral cytokines can cross the blood-brain barrier through fenestrated brain capillaries in the choroid plexus, organum vasculosum of the lamina terminalis, median eminence, subfornical organ, and area postrema [10]. Moreover, it was described that, in response to peripheral bacterial endotoxin, proinflammatory IL-1*β* could be synthesized by macrophage-like cells in the circumventricular organs and choroid plexus, where the blood-brain barrier is deficient [11]. During inflammation, the cytokines may be also transported into the brain via blood-brain barrier by saturated, self-inhibitable transport mechanisms [12]. However, significant sources of central proinflammatory cytokines may generate their own synthesis within the brain tissue including hypothalamus [9, 13]. This brain synthesis of proinflammatory cytokines could be induced by a fast neural pathway [11]. It was shown that the electrical stimulation of the vagus nerve induces the expression of brain IL-1, and vagotomy abrogates the induction of expression of brain IL-1 in response to intraperitoneal lipopolysaccharide [14, 15]. The synthesis of proinflammatory cytokines in brain structures may be also stimulated and controlled by the same cytokines originating from the periphery or produced by the circumventricular organs and choroid plexus. This is because proinflammatory cytokines amplify their own synthesis and secretion [16]. However, an important role in the transmission and amplification of inflammatory signals in the brain may cause auto- and paracrine stimulation of proinflammatory cytokines in the brain tissue. This autostimulatory effect of IL-1 on its synthesis was observed in many cytokine-secreting cells including microglia [17, 18]. It is well established that central proinflammatory cytokines could directly affect the processes regulated at the level of hypothalamus via their corresponding receptors [19]. Acting centrally proinflammatory cytokines, especially IL-1*β*, are considered to be important modulator of endocrine system. At the level of hypothalamus IL-1*β* was found to stimulate the hypothalamic-pituitary-adrenal stress axis [20]. On the other hand, the injection of exogenous IL-1*β* into the region of hypothalamus suppressed secretion of gonadoliberine (GnRH) leading to downstream inhibition of the hypothalamic-pituitary-gonadal axis activity [21, 22]. Therefore, the reduction of proinflammatory cytokines synthesis in the hypothalamic area may profoundly attenuate the central response on the peripheral inflammation.

The study was designed to determine the effect of peripheral injection of SB203580 on the synthesis of IL-1*β*, IL-6, and TNF*α* in the hypothalamus of ewes during prolonged inflammation. SB203580 is pyridinyl imidazole compound that is a selective ATP-competitive inhibitor of the p38 mitogen-activated protein kinase (MAPK). It has previously been established that SB 203580 acts primarily to block the catalytic activity of p38 MAPK but not its activation by upstream MAPK [23]. However, it is worth mentioning that biochemical analysis of SB203580 activity showed that this inhibitor lost its specificity when it was used at high concentration and could also suppress activity of other kinases such as lymphocyte kinase, glycogen synthase kinase 3, and protein kinase B [24]. The p38 MAPK is an important mediator of stress-induced gene expression. In particular, the p38 kinase is known to play a key role in LPS-induced signal transduction pathways leading to cytokine synthesis [25]. Previous studies indicated that p38 MAPK is involved in the inflammatory-dependent upregulation of proinflammatory cytokines synthesis at the translational and posttranslational levels [23, 26]. It was shown that SB203580 strongly inhibited LPS-stimulated synthesis of IL-6 and TNF-*α* in hepatic stellate cells [27].

## 2. Materials and Methods

### 2.1. Animals

The studies were performed on adult, 3-year-old Polish mountain sheep in the anestrous season (April-May). All animals were in good condition. The body condition of all animals was estimated at three- on a five-point scale. The animals were maintained indoors in individual pens and exposed to natural daylight. The ewes were well adapted to the experimental conditions and always had visual contact with neighbouring ewes, even during the experimental period. This avoided the stress of social isolation. The animals were fed a constant diet of commercial concentrates with hay and water constantly available.

All procedures on animals were performed with the consent of the Local Ethics Committee of the Warsaw Agriculture University.

### 2.2. Experimental Procedures

Venous catheters were implanted into the jugular vein on the day prior to the experiment. The ewes (*n* = 24) were randomly divided into four experimental groups: group I—control (*n* = 6); group II—SB203580 treated (*n* = 6); group III—LPS-treated (*n* = 6); group IV—LPS- and SB203580 treated (*n* = 6). In treated animals, the prolonged immune stress was induced by the intravenous (i.v.) administration of LPS from* Escherichia coli 055:B5* (Sigma, St. Louis, MO, USA) dissolved in saline (0.9% w/v NaCl) (Baxter, Deerfield, IL, USA) at a concentration of 10 mg/L into the jugular vein (400 ng/kg). For six days, the animals received a single injection of LPS or saline at 8 am. On day 7 of the experiment, 30 mins prior to saline/LPS treatment, ewes from groups II and IV received i.v. injection of SB203580 hydrochloride (500 *μ*g/kg) (Axon Medchem BV, Groningen, Netherlands). Concurrently, the animals from the control and LPS treated groups received 2 mL of saline injection. Animals were euthanized three hours after the LPS/saline injection and the brains were rapidly removed from the skulls. The blocks of brains encompassing hypothalamus were sectioned sagittally and dissected from both sides according to stereotaxic atlas of the sheep brain [28]. Landmarks were the mammillary body, median eminence, and optic chiasm. The depth of the cuts was 2.5 to 3 mm. Following collection, all tissue was divided into two and then frozen in liquid nitrogen and stored at −80°C.

### 2.3. ELISA Assay for IL-1*β*, IL-6, and TNF*α* Concentration in the Hypothalamus

The concentrations of IL-1*β*, IL-6, and TNF*α* in the hypothalamus were determined using a commercial IL-1*β*, IL-6, and TNF*α* ELISA kit (BlueGene Biotech CO., LTD., China) designed for sheep. The tissues were homogenized in 1 mL of cold phosphate buffered saline (0.02 M); then homogenates were subjected to two freeze-thaw cycles to further break the cell membranes. Homogenates were then centrifuged for 15 min at 1500 ×g at 4°C. The supernatants were aliquoted and stored until assay at −80°C. All steps in the assays were performed according to the manufacturer's instructions. The incubation of plates and absorbance measurement at 450 nm were performed using a VersaMax reader (Molecular Devices LLC., Sunnyvale, California, USA). The sensitivity for all assays was 1.0 pg/mL. The significance of the differences in the levels of IL-1*β* between the experimental groups was assessed by the Mann-Whitney *U* test. Statistical significance was defined as *P* < 0.01.

### 2.4. Determining the Relative Gene Expression

Total RNA from hypothalamus was isolated using NucleoSpin RNA II Kit (MACHEREY-NAGEL Gmbh & Co; Düren, Germany) according to a manufacturer's instruction. The purity and concentration of isolated RNA were spectrophotometrically quantified by measuring the optical density at 230, 260, and 280 nm in a NanoDrop 1000 instrument (Thermo Fisher Scientific Inc., Waltham, USA). The RNA integrity was verified by electrophoresis using 1% agarose gel stained with ethidium bromide. Maxima First Strand cDNA Synthesis Kit for RT-qPCR (Thermo Fisher Scientific Inc., Waltham, USA) was used to prepare cDNA synthesis. As a starting material for this PCR synthesis 2 *μ*g of total RNA was used.

Real-time RT-PCR was carried out using HOT FIREPol EvaGreen qPCR Mix Plus (Solis BioDyne, Tartu, Estonia) components and HPLC-grade oligonucleotide primers synthesised by Genomed (Poland). Specific primers for determining the expression of housekeeping genes and the genes of interest were designed using Primer 3 software (Table 1). One tube contained 4 *μ*L PCR Master Mix (5x), 14 *μ*L RNase-free water, 1 *μ*L primers (0.5 *μ*L each, working concentration was 0.25 *μ*M), and 1 *μ*L cDNA template. The tubes were run on the Rotor-Gene 6000 (Qiagen, Duesseldorf, Germany). The following protocol was used: 95°C in 15 mins for activating Hot Star DNA polymerase and finally the PCR including 30 cycles at 95°C in 10 sec for denaturation, 60°C in 20 sec for annealing, and 72°C in 10 sec for extension. After the cycles, a final melting curve analysis under continuous fluorescence measurements was performed to confirm the specificity of the amplification.

Relative gene expression was calculated using the comparative quantification option of Rotor Gene 6000 software 1.7. (Qiagen, Duesseldorf, Germany). The second differential maximum method [29] was used to calculate reaction efficiencies. A set percentage of the maximum fluorescence value was used to calculate the beginning of the exponential phase. To compensate for the variation in cDNA concentrations and the PCR efficiency between tubes, an endogenous control gene was assayed in each sample and used for normalization. Initially, four housekeeping genes,* GAPDH, *β*-actin, PPIC,* and* HDC1, *were tested. The BestKeeper was used to determine the most stable housekeeping gene for normalizing genes of interest expression. The BestKeeper is based on the pair-wise correlation analysis of all pairs of candidate genes [30] and calculates variations of all reference genes SD (±Ct). GAPDH was selected as the best endogenous control gene. It had the lowest SD (±Ct) value and correlation coefficient with the remaining analyzed housekeeping genes.

The results are presented as a relative gene expression of the target gene versus housekeeping gene, relative expression value, and mean ± S.E.M. The average relative quantity of gene expression in control groups was set to 1.0. The significance of differences between the experimental groups was assessed by the Mann-Whitney *U* test. Statistical significance was defined as *P* < 0.01.

## 3. Results

### 3.1. Influence of SB203580 and Prolonged LPS Treatment on IL-1*β*, IL-6, and TNF*α* Synthesis in the Hypothalamus

**Figure 1:**
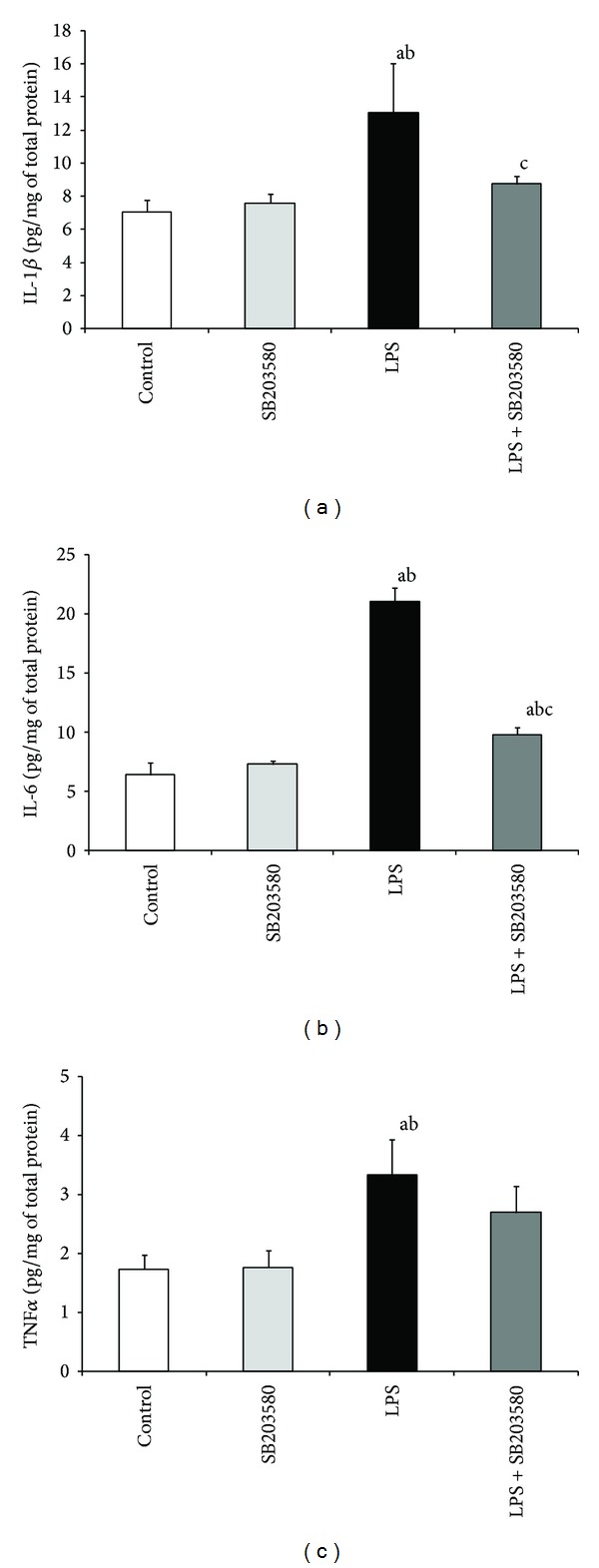
Effect of lipopolysaccharide (LPS) (400 ng/kg; i.v./7 days) and SB203580 (500 *μ*g/kg) injections on IL-1*β* (a), IL-6 (b), and TNF*α* (c) expression in the hypothalamus on day 7 of the experiment. a, b, c—*P* < 0.01 (indicate values that differ significantly from the control, SB203580 control, and LPS treated groups, resp., according to the Mann-Whitney *U* test). Data are presented as a mean value ± S.E.M.

Sevenfold administrations of LPS increased (*P* < 0.01) the amount of all analyzed proinflammatory cytokines in the hypothalamus compared with both control groups. SB203580 decreased (*P* < 0.01) LPS-induced elevation of IL-1*β* and IL-6 concentration. However, in the group with concomitant SB203580 and LPS treatment the level of IL-6 stayed increased (*P* < 0.01) compared with control groups. On the other hand, the injection of SB203580 did not affect the endotoxin-stimulated TNF*α* expression in the hypothalamus (Figure 1).

### 3.2. Effect of SB203580 and Prolonged LPS Treatment on IL-1*β*, IL-6, TNF*α*, and Their Corresponding Receptors Gene Expression in the Hypothalamus

**Figure 2:**
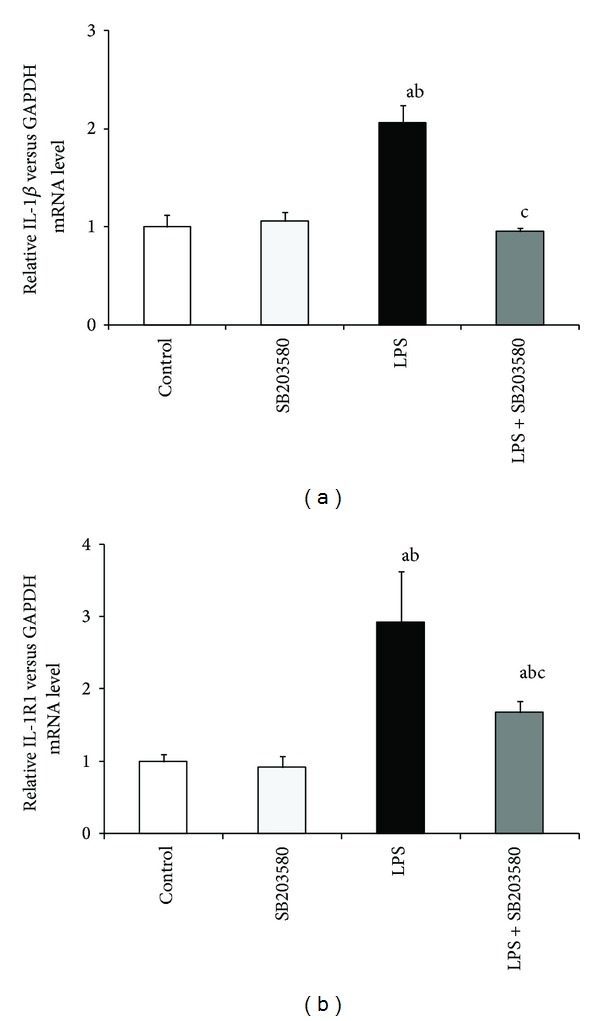
Influence of lipopolysaccharide (LPS) (400 ng/kg; i.v./7 days) and SB203580 (500 *μ*g/kg) injections on the gene expression of IL-1*β* (a) and IL-1R1 (b) in the hypothalamus on day 7 of the experiment. a, b, c—*P* < 0.01 (indicate values that differ significantly from the control, SB203580 control, and LPS treated groups, resp., according to the Mann-Whitney *U* test). Data are presented as a mean value ± S.E.M.

**Figure 3:**
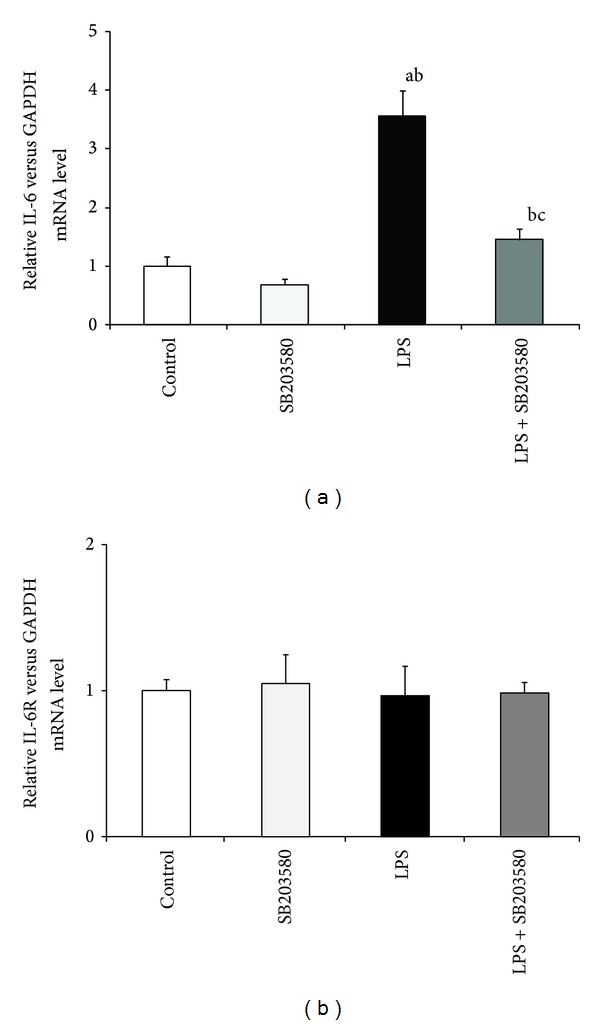
Effect of lipopolysaccharide (LPS) (400 ng/kg; i.v./7 days) and SB203580 (500 *μ*g/kg) injections on the gene expression of IL-6 (a) and IL-6R (b) in the hypothalamus on day 7 of the experiment. a, b, c—*P* < 0.01 (indicate values that differ significantly from the control, SB203580 control, and LPS treated groups, resp., according to the Mann-Whitney *U* test). Data are presented as a mean value ± S.E.M.

**Figure 4:**
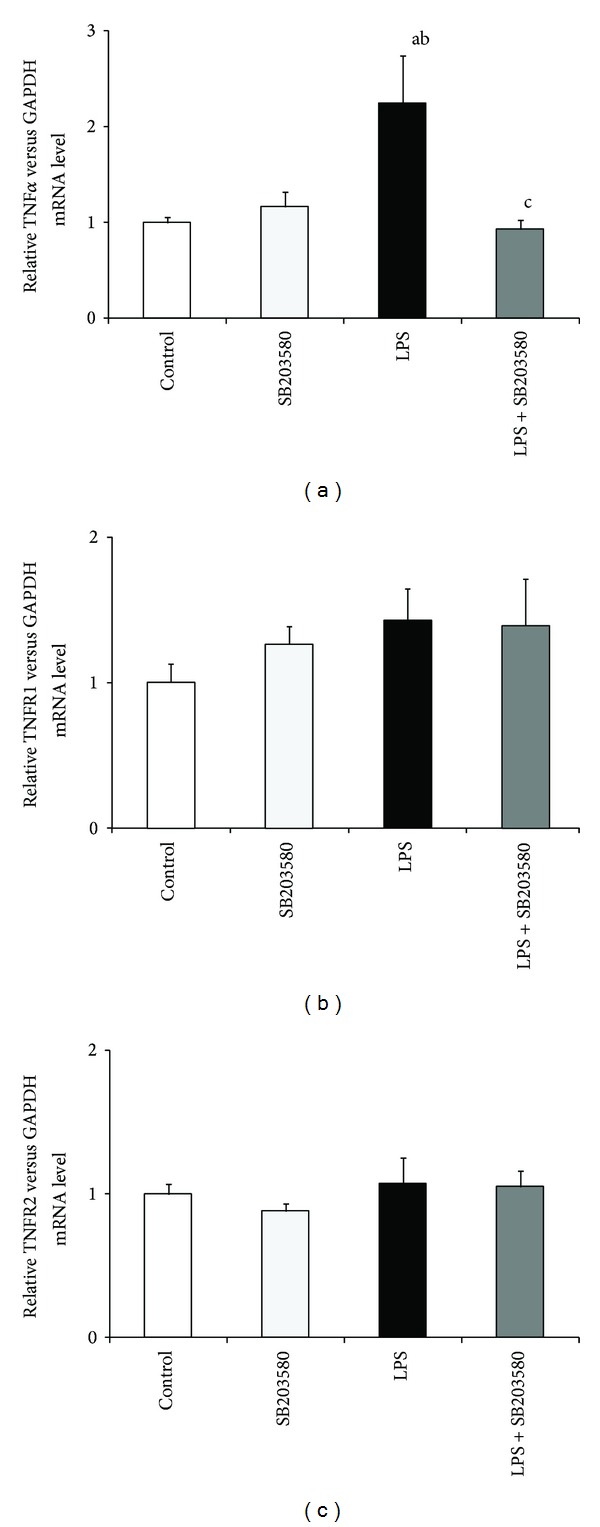
Effect of lipopolysaccharide (LPS) (400 ng/kg; i.v./7 days) and SB203580 (500 *μ*g/kg) injections on the gene expression of TNF*α* (a), TNFR1 (b), and TNFR2 (c) in the hypothalamus on day 7 of the experiment. a, b, c—*P* < 0.01 (indicate values that differ significantly from the control, SB203580 control, and LPS treated groups, resp., according to the Mann-Whitney *U* test). Data are presented as a mean value ± S.E.M.

Seven days of LPS administration simulated (*P* < 0.01) the gene expression of* IL-1*β** (Figure 2)*, IL-6 *(Figure 3), and* TNF*α** (Figure 4) in the hypothalamus. On the other hand, prolonged endotoxin treatment stimulated (*P* < 0.01) only the expression of IL-1R1 gene but had no effect on IL-6R, TNFR1, and TNFR2 mRNA level in the hypothalamus. SB203580 reduced (*P* < 0.01) LPS-dependent elevation of IL-1*β*, IL-6, and TNF*α* as well as IL-1R transcription. However, SB203580 did not completely abolish the effect of inflammation on the gene expression of TNF*α* and IL-1R1 which stayed increased (*P* < 0.01) compared to the control groups.

## 4. Discussion

Our study demonstrated that prolonged inflammation induced through 7 days of LPS treatment elevated local synthesis of IL-1*β*, IL-6, and TNF*α* in the hypothalamus of sheep. The strongest stimulatory effect of inflammation was determined in the case of IL-6, which showed the highest relative increase in the concentration. These results fully support the data obtained in previous studies with single and repeated LPS injections which reported the potentiation of proinflammatory cytokines synthesis in the brain of mice [6, 31]. They also determined higher elevation of IL-6 mRNA levels compared with IL-1*β* and TNF*α* in response to peripheral inflammatory stimuli. This could have a profound effect on hypothalamic activity. It was concluded that central proinflammatory cytokines, especially IL-1, are able to induce changes in the brain neurochemistry. These cytokines induce norepinephrine release in the brain (most markedly in the hypothalamus), increase brain concentrations of tryptophan and the metabolism of serotonin, decrease acetylcholine secretion, and provoke modest changes in brain dopamine [32]. Among others acting at the level of hypothalamus, proinflammatory cytokines have been identified as being responsible for stimulation of thermogenesis [33], the hypothalamic-pituitary-adrenal axis activation [34, 35], and the hypothalamic-pituitary-gonadal axis inhibition [21]. These actions of proinflammatory cytokines in the hypothalamus enable the existence of their corresponding receptors in this region of the brain [19]. The expression of their receptor was determined in microglia, astrocytes, and even neurons [36–39]. In our study prolonged inflammation elevated only IL-1 type I (R1) gene expression but did not influence the transcription of cytokines receptor: IL-6R, TNFR1, and TNFR2 in the hypothalamus. The stimulatory effect of endotoxin treatment on IL-1R1 expression in the brain has been previously described in sheep [8, 40] and mice [41, 42]. This could lead to the assumption that in the hypothalamus peripheral inflammation increases the sensitivity of IL-1R1 expressing cell on the action of IL-1*β*. Although, no affect of peripheral inflammatory stimuli on the gene expression of IL-6R was found in the hypothalamus, it should be pointed out that the amount of mRNA for IL-6 corresponding receptor was the highest among transcripts encoding other analyzed cytokines receptors. Therefore, based on our data it is impossible to judge which cytokine is pivotal in the transmission of the inflammatory effects in this region of the hypothalamus.

The p38 MAPK is one of the three groups of mitogen-activated protein kinases which are key enzymes in the signal transduction cascade from the extracellular environment to the nucleus of essentially every eukaryotic cell type [43]. The p38 MAPKs are strongly activated* in vivo* by environmental stresses and inflammatory cytokines but less by serum and growth factors. Therefore, together with the JNK family, p38 MAPKs are also known as stress-activated protein kinases [44]. The p38 MAPK was first recognized for its role in inflammation in regulating the biosynthesis of proinflammatory cytokines such as IL-1 and TNF*α* in endotoxin-stimulated monocytes [26]. Subsequently it was also found to be involved in regulating the production of IL-8 in response to IL-1*α* and IL-1*β* [45] and the production of IL-6 in response to IL-1 and TNF*α* [43, 46]. The results of the* in vitro* studies indicate that p38 MAPK inhibition by SB203580 strongly reduces the IL-1*β* induced synthesis of IL-6 and indicates that a key element of p38-dependent IL-6 regulation occurs at the level of mRNA stability [43]. The* in vitro* experiments also demonstrate that SB203580 inhibits at the dose-dependent manner endotoxin-stimulated expression of IL-1*β* in the monocytic cell lines [25]. It should be noted that the results of the study performed on rat primary cortical glial cell cultures indicate that inhibition of p38 MAPK by SB203580 did not prevent LPS and IL-1*β* induced expression of IL-1*β* but did inhibit the IL-1*β*-induced expression of c-fos and inducible nitric oxide synthase [47]. It is worth noticing that the anti-inflammatory effect of SB203580 has been also described in the* in vivo *study. In the experiment carried out on mice and rat, treatment with SB203580 for 30 min prior to the injection of bacterial endotoxin suppressed the plasma level of TNF*α* at the dose-dependent manner [48]. In the same study, SB203580 also inhibited the circulating concentration of both IL-6 and TNF*α* in rat and mice with experimentally induced arthritis. Moreover, treatment with SB203580 reduced mortality in a murine model of endotoxin-induced shock. Anti-inflammatory effects of SB203580 and its possible therapeutic use were also determined in the mice with experimentally induced endometriosis. It was shown that repeated for 24-day injection of SB203580 strongly reduced the synthesis of IL-1*β* and TNF*α* translation in the mice endometrium [49].

Our study also demonstrated that i.v. injection of SB203580 attenuates endotoxin-dependent synthesis of the proinflammatory cytokines in the hypothalamus. The peripheral administration of the p38 MAPK inhibitor restored synthesis of IL-1*β* to the control value and reduced IL-6 expression in the hypothalamus. However, the effect of SB203580 on the synthesis of hypothalamic TNF*α* was observed only in the case of transcriptomic analysis. The lack of effect of p38 MAPK inhibition on TNF*α* synthesis at the level of protein expression could be connected with relatively low elevation of this cytokine in the hypothalamus and limited sensitivity of ELISA assay. However, it should be emphasized that the effectiveness of anti-inflammatory treatment did not require blockade of all proinflammatory cytokines. There is evidence that cytokines exist in “cascades” and that interrupting one cytokine interrupts the cascade [50]; for example, blocking TNF*α* reduces the activity of IL-6 and IL-1*β* [51] while blocking IL-1*β* reduces IL-6 [52].

Our study determined that changes in the synthesis of cytokines and their receptors after peripheral administration of SB203580 do not have to be an effect of direct inhibition of p38 MAPK in the hypothalamic tissue. It is possible that SB203580 attenuates the peripheral inflammatory response reflecting on reduced inflammatory signal and reduced proinflammatory cytokines synthesis in the hypothalamus. The study conducted on mice demonstrated that reduction of the peripheral inflammatory response using the anti-inflammatory drug which did not cross the blood-brain barrier was enough to suppress the brain synthesis of IL-1*β* [53]. This suggested that even a relatively small reduction of peripheral cytokines, particularly IL-1*β*, may translate into greater attenuation of cytokines synthesis in the brain. This may be because LPS-induced production of IL-1*β* in the periphery must exceed a fairly high threshold to increase the levels of IL-1*β* in the brain [53]. One of the endogenous mechanisms controlling the synthesis of proinflammatory cytokines is effect of choline. It was reported that direct stimulation of the vagus nerve or pharmacological inhibition of acetylcholinesterase activated the cholinergic anti-inflammatory pathway which suppressed systemic production of cytokines such as TNF*α*, IL-1, and IL-6 [54]. The studies carried out on rats showed that intrathecal injection of SB203580 caused a 3-fold rise in the high-frequency power spectral component of heart rate variability which is a widely used parameter of parasympathetic activity that directly correlates with activation of the cholinergic anti-inflammatory pathway [55]. Thus, the ability of SB203580 inhibitors to activate the vagus nerve could at least partially explain observed reduction of proinflammatory cytokines synthesis in our study.

The findings that inhibiting p38 MAPK blocks LPS-induced proinflammatory cytokine production seem to initiate new perspectives in the treatment of chronic inflammatory diseases also in the central nervous system. However, recent studies suggest that the role of p38 MAPK during inflammation may be more complex and ambiguous [56]. It was reported that p38 MPAK is also involved in the activation of anti-inflammatory processes. In myeloid cells activation of p38 MAPK signaling pathway limited inflammation in a UV-induced irradiation model [57]. This immunomodulatory effect of p38 MAPK seems to be mediated by the induction of the anti-inflammatory cytokine IL-10 and the inhibition of proinflammatory IL-12 [56–59]. It should be noted that the results of* in vitro* study on primary human monocytes demonstrated that SB203580 produced profound inhibition of spontaneous production of IL-1 and TNF*α*. Unfortunately, it was also determined that SB203580 significantly increases LPS-stimulated IL-6 production and decreases the synthesis of anti-inflammatory IL-10 in primary human monocytes [60]. These potential proinflammatory effects of SB203580 treatment suggest that all therapy using p38 MAPK inhibitors should be performed cautiously and with analysis conducted on expected and unexpected consequences of the treatment.

## Figures and Tables

**Table 1:** All genes analyzed by real-time PCR are listed with their full name and abbreviation.

GenBank acc. number	Gene	Amplicon size [bp]	Forward/reverse	Sequence 5′→3′
NM_001034034	***GAPDH*** Glyceraldehyde-3-phosphate dehydrogenase	134	Forward Reverse	AGAAGGCTGGGGCTCACT GGCATTGCTGACAATCTTGA

U39357	***ACTB*** Beta actin	168	Forward Reverse	CTTCCTTCCTGGGCATGG GGGCAGTGATCTCTTTCTGC

NM_001076910	***PPIC*** Cyclophilin C	131	Forward Reverse	ACGGCCAAGGTCTTCTTTG TATCCTTTCTCTCCCGTTGC

BC108088.1	***HDC1*** *Bos taurus* histone deacetylase 1	115	Forward Reverse	CTGGGGACCTACGGGATATT GACATGACCGGCTTGAAAAT

X54796.1	***IL-1*** **β** Interleukin-1 beta	137	Forward Reverse	CAGCCGTGCAGTCAGTAAAA GAAGCTCATGCAGAACACCA

NM_001206735.1	***IL-1R1*** Interleukin-1 receptor, type I	124	Forward Reverse	GGGAAGGGTCCACCTGTAAC ACAATGCTTTCCCCAACGTA

NM_001009392.1	***IL-6*** Interleukin-6	165	Forward Reverse	GTTCAATCAGGCGATTTGCT CCTGCGATCTTTTCCTTCAG

NM_001110785	***IL-6R*** Interleukin 6 receptor	149	Forward Reverse	TCAGCGACTCCGGAAACTAT CCGAGGACTCCACTCACAAT

NM_001024860	***TNF*** **α** Tumor necrosis factor alpha	153	Forward Reverse	CAAATAACAAGCCGGTAGCC AGATGAGGTAAAGCCCGTCA

NM_174674	***TNFR1*** Tumor necrosis factor receptor superfamily, member 1A	137	Forward Reverse	AGGTGCCGGGATGAAATGTT CAGAGGCTGCAGTTCAGACA

NM_001040490	***TNFR2*** Tumor necrosis factor receptor superfamily, member 1B	122	Forward Reverse	ACCTTCTTCCTCCTCCCAAA AGAAGCAGACCCAATGCTGT
